# Effect of Traditional Stir-Frying on the Characteristics and Quality of Mutton Sao Zi

**DOI:** 10.3389/fnut.2022.925208

**Published:** 2022-06-23

**Authors:** Shuang Bai, Liqin You, Yongrui Wang, Ruiming Luo

**Affiliations:** ^1^Beijing Advanced Innovation Center for Food Nutrition and Human Health, Beijing Technology and Business University, Beijing, China; ^2^School of Food and Wine, Ningxia University, Yinchuan, China; ^3^College of Biological Science and Engineering, North Minzu University, Yinchuan, China

**Keywords:** mutton, stir-frying, Maillard reaction products (MRPs), polyaromatic hydrocarbons (PAHs), heterocyclic aromatic amines (HAAs)

## Abstract

The effects of stir-frying stage and time on the formation of Maillard reaction products (MRP) and potentially hazardous substances with time in stir-fried mutton sao zi were investigated. Furosine, fluorescence intensity, Nε-(1-carboxymethyl)-L-lysine (CML), Nε-(1-carboxyethyl)-L-lysine (CEL), polyaromatic hydrocarbons PAHs), heterocyclic aromatic amines (HAAs), and acrylamides (AA) mainly presented were of stir-fried mutton sao zi. The furosine decreased after mixed stir-frying (MSF) 160 s due to its degradation as the Maillard reaction (MR) progressed. The fluorescent compound gradually increased with time during the stir-frying process. The CML and CEL peaked in MSF at 200 s. AA reached its maximum at MSF 120 s and then decreased. All the 5 HAAs were detected after MSF 200 s, suggesting that stir-frying mutton sao zi was at its best before MSF for 200 s. When stir-frying exceeded the optimal processing time of (MSF 160 s) 200 s, the benzo[a]pyrene peaked at 0.82 μg/kg, far lower than the maximum permissible value specified by the Commission of the European Communities. Extended stir-frying promoted MRP and some hazardous substances, but the content of potentially hazardous substances was still within the safety range for food.

## Introduction

Compared with other meats, mutton has a special flavor, tender texture, and unique sensory attributes, so it is eaten by more and more people globally, especially in Asia and Europe (1). In recent years, more and more scholars have concentrated on the safety of mutton products in the process of processing. The research on mutton products mainly focuses on mutton sausages (2), mutton ham (3), fried mutton (4), and roasted mutton (5), but only a few reports have been published on the research of stir-fried mutton products.

Mutton sao zi is one of the well-known traditional stir-fried mutton product in northwest China due to the tender texture, special sensory attributes, and flavor, and so it has gained popularity among people (6). The cooking of lamb meat can improves the safety of lamb meat by killing or inhibiting microbial reproduction and improving physicochemical parameters, such as color and texture (7).

As Wang et al. (8) have reported the lipid oxidation, Maillard reaction (MR), peptides, amino acids, and thermal degradation of thiamine also contribute to the unique flavor and quality during the high-temperature stir-frying of meat. The generation of Maillard reaction products (MRPs) depends on the basic composition of processed products, processing temperature, and time (9). Although there are numerous publications on the MRPs of mutton, what lacks is information about the generation of mutton sao zi MRPs during stir-frying. How to judge the MR process? Trevisan et al. (9) evaluated MR progresses through the compounds generated by the process of MR. Furosine is formed in the first stages of MR, as well as an indirect measure index formed from the acid hydrolysis of Amadori products (10). At the same time, the fluorescent substances are generated by dehydration and fission of Amadori products during the MR process. Therefore, it is an effective method to evaluate the degree of MR in food by measuring the increase or decrease of absorbance values at 280 and 420 nm (11). As the MR progresses, Nε-(1-carboxymethyl)-L-lysine (CML) and Nε-(1-carboxyethyl)-L-lysine (CEL) are generated in the later stage of MR (12, 13). Furthermore, the formation of AGEs is promoted by active amino acids and carbonyl compounds (14).

However, some harmful substances may be produced when meat products are cooked at high temperatures for long periods, such as generating AA, HAAs, and polyaromatic hydrocarbons (PAHs), which will lower the flavor and quality of cooked mutton and cause harmful effects on the consumers’ health (1). AA is mainly generated by reducing sugars and free asparagine during high-temperature processing, a potential carcinogen and a mutagenic agent, also another important MRP, which can have serious effects on human health (9). Research of the pathway, stage, and time of AA formation in stir-fried mutton sao zi is important to reducing the AA content. The generation of PAHs in meat products could be affected by the processing temperature, cooking method (smoking, roasting, baking, frying, grilling), antioxidants, and meat composition (15, 16). The extent to which HAAs are produced in cooked meats depends on the type of meat, cooking temperature, cooking time, and the degree of MR during cooking (17). The formation of potentially hazardous substances can be mitigated by adjusting thermal processes or process conditions. It is therefore very important to choose the optimal process parameters and reduce the formation of these harmful compounds during the stir-frying of mutton sao zi.

At present, there are few studies on MRP and potentially hazardous substances in the process of stir-frying mutton sao zi. The basic components, MRP, and potentially hazardous substances were determined and analyzed in stir-frying of mutton sao zi during this study, and their safety was evaluated. The MRP and potentially hazardous substances generated in this process were analyzed with an assessment of the safety considerations. The purpose of this work is to analyze the MR change process of industrial stir-fried mutton sao zi, reduce the hazardous substances produced in the process of stir-frying, and assure the safety of industrial stir-fried mutton sao zi.

## Materials and Methods

### Materials and Reagents

The standards of CML, CEL, Nε-(1-carboxymethyl)-L-lysine-d4 (d_4_-CML), and Nε-(1-carboxyethyl)-L-lysine-d4 (d_4_-CEL) were obtained commercially from Toronto Research Chemicals (North York, ON, Canada). Then 1 mL of furosine dihydrochloride solution (100 μg/mL) was purchased from Alta Technology Co., LTD (Tianjin, China). The 16 mixed standards of PAH were obtained from MANHAGE Biotechnology Co., LTD (Beijing, China). The HAAs standards used were 2-amino-3-methyl-3H-imidazo[4,5-f]quinoline (IQ), 2-amino-3,8-dimethylimidazo[4,5-f]quinoxaline (MeIQx), 2-amino-3,4,8-trimethylimidazo[4,5-f]quinoxaline(4,8-DiMeIQx), 2-amino-1-methyl-6-phenylimidazo [4,5-b]pyridine (PhIP), 2-amino-9H-pyrido[2,3-b]indole (AαC), 2-amino-3-methyl-9H-pyrido[2,3-b]indole (MeAαC), 3-amino-1-methyl-5H-pyrido[4,3-b]indole (Trp-P-2), 2-amino-3,4,7,8-tetramethyl-3H-imidazo[4,5-f]quinoxaline (4,7,8-TriMeIQx), and 1-methyl-9H-pyrido[3,4-b]indole (harman), and 9H-pyrido-[3,4-b]indole (norharman). The purity of the HAAs standards (IQ, MeIQx, PhIP, 4,8-DiMeIQx, PhIP, AαC, MeAαC, Trp-P-2, Norharman, and Harman) were greater than 99% and purchased from Yuanye Bio-Technology Co. (Shanghai, China). Each standard requires a solution of acetonitrile–water (5:95 v/v) that is used for further dilution and 4,7,8-TriMeIQx was used as an internal standard solution. Standard acrylamide (≥99.9%) was obtained from Baolaibo Technology Co., LTD (Beijing, China) and standard ^13^C_3_-acrylamide (LC-MS, ≥99.9%) was from Isotope Laboratory (Cambridge, United Kingdom). The 16 PAH mixed standards (MIX-16, 200 μg/mL) of naphthalene, benzo[a]pyrene, acenaphthylene, benzo[k]fluoranthene, acenaphthene, fluorene, phenanthrene, anthracene, fluoranthene, pyrene, benz[a]anthracene, chrysene, benzo[b]fluoranthene, indeno[1,2,3-cd]pyrene, dibenzo[a,h]anthracene, benzo[ghi]perylene were purchased from MANHAGE Biotechnology Co., LTD (Beijing, China).

### Preparation of Samples

Fresh tail fat (approximate composition: 8.34% water, 3.25% protein, 82.21% lipids, and 1.94% ash) and hind leg meat with surface fat removed (approximate composition: 73.08% water, 19.60% protein, 3.01% lipids and 1.86% ash) were obtained from a commercial meat company (Yanchi, China). The sample and stir-frying conditions are the same as the previous articles published by our laboratory (6). To study the influence of PAHs and HAAs on stir-fried mutton sao zi, 4 treatment groups were added after mixed stir-frying (MSF) 200 s.

### Determination of the Basic Components

The pH values were determined by a plug-in portable pH meter (205-Y60, Testo Instruments International Trade Co., Ltd., Germany). Chrominance value (L*, a*, b*) was determined based on CIE D65 standard light source on a CR-400 Konica Minolta (Beijing, China). The texture profile analysis (TPA) and shear force values of stir-fried mutton sao zi were determined by a texture analyzer TA-XT2i (Stable Micro Systems LTD., England). The measurement parameters of TPA texture analysis are as follows: the speed before the test, 1.00 mm/s; the speed during the test, 1.00 mm/s; the speed after the test, 5.0 mm/s; the probe, P35, the time interval between two pressings is 5 s, trigger point load, 5 g; compression ratio, 50%. The shear-force measurement parameters are set as follows: pre-measurement rate, 2.0 mm/s; mid-measurement rate, 2.0 mm/s; post-measurement rate, 10.0 mm/s; distance, 30.0 mm; trigger force, 20 g; probe HDP-BSW, shear perpendicular to the direction of muscle fibers of each sample was measured 3 times, and the average value was taken. Cooking loss = (before cook weight–after cook weight)/before cook weight * 100% (18).

### Determination of Thiobarbituric Acid Reactive Substances

According to Fan et al. (19), the content of thiobarbituric acid reactive substances (TBARS) in sample were determined by CN61M-752 spectrophotometer (Haifuda Instrument Co., LTD.Beijing, China). The results of TBARS value were expressed as milligrams of malondialdehyde (MAD) per kg sample. Briefly, 25 mL of 20% (w/w) trichloroacetic acid solution and 20 mL of chilled deionized water were added to the ground meat (5 g), homogenized for 30 s at 10,000 r/min using a homogenizer, and allowed to stand for 20 min at room temperature. Then, the mixture was centrifuged at 6,000 × *g* for 10 min, and the supernatant was diluted to 50 mL with deionized water. Finally, 5 ml of the diluted supernatant was mixed with 0.02 M aqueous solution of 2-thiobarbituric acid (5 ml) in a stoppered test tube and kept at 95°C for 30 min in a water bath. After the reaction solution was cooled to room temperature by flowing water, the absorbance values of the filtrate were read at 532 nm using a CN61M-752 spectrophotometer with deionized water as the blank. The content of TBARS was expressed as mg MAD/kg meat according to the following formula:


TBARS=A532×7.8

where A_532_ is the absorbance at 532 nm with a 10-mm cuvette.

### The Changes of Maillard Reaction Products

#### Determination of Furosine

According to Bayrak Kul et al. (10), furosine was determined with minor modifications. A total of 100 mg of freeze-dried sample was hydrolyzed with 2 mL of 8 N HCl at 110°C for 23 h in a vial. Before sealing, the ampoule bottle was filled with nitrogen for 2 min. The samples were filtered with a 0.22 μm PVDF membrane after cooling at 25°C. Following the recommendation of Delgado-Andrade et al. (11), a 0.5 mL aliquot of the hydrolyzate was freeze-dried and dissolved in a mixture of 1 mL of water, acetonitrile, and formic acid (95:5:0.2, v:v:v). The analysis was carried out using LC-20A HPLC system (Shimadzu, Japan) with a diode array detector, followed by a separation which was achieved on a C_18_ column (150 mm × 4.6 mm × 5 μm, Shim-pack GIST) at 40°C. With an injection volume of 10 μL, the detection was carried out at 280 nm. The study adopted a mobile phase A of 0.4% acetic acid. The elution was isocratic with a flow rate of 0.8 mL/min and a retention time of about 5 min. Furosine was quantified by the external standard method. The protein amount was determined according to the AOAC (20). The standard curve was plotted with 1, 2, 5, 10, and 30 μg/mL of furosine standard. The obtained results were given in mg/100 g of protein.

#### Determination of Fluorescent Compounds and Absorbance

Fluorescent compounds (FI) were determined according to Trevisan et al. (9). The absorbance at 280 and 420 nm was represented the early intermediate and intermediate products of MRPs in stir-frying mutton sao zi samples, detected using a 960 MC fluorospectro photometer (Precision Instrument Co., Ltd, Shanghai, China).

#### Determination of Nε-(1-Carboxymethyl)-L-Lysine and Nε-(1-Carboxyethyl)-L-Lysine

According to the method of Yu et al. (21) with minor modifications, the CML and CEL values of stir-frying samples were determined. Briefly, the CML and CEL were prepared following the steps below. A total of 5 mg of freeze-dried and pulverized stir-fried mutton sao zi were accurately weighed and degreased three times with 5 mL of n-hexane, then added to 1 mL of sodium borohydride solution and 1.5 mL of borate buffer solution (0.2 M, pH 9.2) and stored at 4°C for 10 h in the refrigerator. Then 3 mL of concentrated hydrochloric acid (12 mol/L) was added and then acidified in a vial for 24 h at 110°C. The d4-CML and d4-CEL mixed internal standard (150 μL, 1 μL/mL) and 3 mL of water were added when the hydrolyzate was blown dry with nitrogen and let through the MCX cartridge (SPE, Oasis MCX cartridge, 60 mg, 3 cc, 60 μm). With 3 mL of methanol and 0.1 mol/L hydrochloric acid, respectively, the MCX cartridge was washed and activated. Then the sample solution was let through the MCX cartridge, with wash impurities washed with 3 mL of water and 3 mL of methanol successively, and 5 mL of methanol solution with a volume fraction of 5% ammonia water. The collected eluate was concentrated by nitrogen blowing, re-dissolved in 1 mL of methanol–water (80:20, V/V) solution, and after passing through a 0.22-μm PVDF membrane, prepared for injection analysis. Briefly, the separations were performed using a Phenomenex Synergi 4 μ Hydro-RP80A column (250 mm × 2 mm, 4 μm) equipped with a column oven at 35°C. The gradient elution was performed at a flow rate of 0.2 mL/min with eluent A as 5 mmol/L ammonium acetate solution and eluent B as methyl alcohol and injection volume as 10 μL. The detection was conducted using MS with electron spray ionization (ESI) and multiple reaction monitoring. The LC-MS/MS was optimized using a capillary voltage of 3.0 kV, in positive mode ESI at a source temperature of 100°C, and with a desolvation gas temperature of 300°C, a cone voltage of 20 V, and a cone gas flow rate of 50 L/h. The following positive ions were monitored: CML, m/z 205; d4-CML, m/z 209; CEL, m/z 219; d4-CEL, m/z 223. CML and CEL were quantified using an analytical curve containing five levels of CML concentrations (0.01, 0.10, 0.20, 0.40, and 0.80 μg/mL) and spiked with d4-CML and d4-CEL at a final concentration of 0.1 μg/mL. The curves were obtained by plotting the ratio between the areas of the external standard and the internal standard peaks as a function of the external standard concentration, showing good linearity (*R*^2^ ≥0.9995). The LOD for CML and CEL were 3.6 and 1.9 ng/mL, respectively. The LOQ for CML and CEL were 8.6 and 4.5 ng/mL. For CML and CEL, the recovery (%) ranged from 86.0 to 95.59 and 88.2 to 98.17, respectively.

### Determination of Potentially Hazardous Substances

#### Determination of Acrylamides

Analysis of the AA was determined based on the China National Standard method (22) with minor modifications (9). The changes in AA were determined by CAS Test Technical Services Co., Ltd. (Guangzhou, China), an accredited commercial laboratory, using liquid chromatography-tandem mass spectrometry (Shimadzu, Kyoto, Japan). A total of 1 g of sample was homogenized and then added to 10 μL of internal standard of ^13^C_3_-acrylamide (10 mg/L) and 10 mL of UP water, and then vortexed in a vortex meter for 2 min. The supernatant was collected after centrifugation at 4,000 r/m for 10 min. Then 5 mL of n-hexane was added to the supernatant and the vortex meter was shaken for 2 min. Centrifugation was done at 10,000 r/m for 5 min and then the organic phase was removed. Then 5 mL of n-hexane was used to extract the aqueous phase again. The aqueous phase was filtrated by 0.45 μm PVDF membrane and then purified by HLB solid-phase extraction column. The eluate was collected from 5 mL of the filtrate and eluted with 4 mL of 80% methanol aqueous solution, then purified by Bond Elut-Accucat solid-phase extraction column. The sample liquid was concentrated by nitrogen blowing and then dissolved in 1.0 mL formic acid solution (0.1%) by LC-MS for analysis.

The LC-MS platform consisted of a Shimadzu LC-30A system (Kyoto, Japan). The LC separation was manipulated on a Reversed-phase C_18_ column (2.1 mm × 150 mm, 5 μm) with a flow rate of 0.2 mL/min at 26°C. The methanol/0.1% formic acid (10:90) were wielded as mobile phases.

The scan range was from 30 to 500 Da, and the cation electrospray ionization source (ESI +) was used for detection. For optimal conditions, the ionization sources were set as follows: ion spray voltage floating, 3500 V; Taper hole voltage, 40 V; Rf lens 1 voltage, 30.8 V; ion source temperature, 80°C; dissolvent temperature, 300°C; ion collision energy, 6 eV.

#### Determination of Heterocyclic Aromatic Amines

Heterocyclic aromatic amines (HAAs) were extracted from sausage, roasted pork, and barbecued meatballs with the method reported by Dy et al. (23), Wang et al. (24), and Oz (25) with minor modifications. The HAAs were identified and quantified using liquid chromatography-mass spectrometry (LC-MS)-1290 from Agilent (Santa Clara, CA, United States). The stir-fried mutton sao zi sample was ground to a powder with a freezer grinder (LC-TG-24, LICHEN, Shanghai, China) and then 2 g of meat puree was weighed into a 50-ml centrifuge tube.

A total of 2 g (accurate to 0.01 g) of meat mud was weighed and then added to a 50 mL centrifuge tube, joined by 10 mL of 2 mol/L sodium hydroxide solution, and then vortex mixer oscillates for 2 min. Following an ultrasonic extraction for 30 min, the solution and 12 g of diatomite were filled into the Bond Elut extraction column. The eluent was eluted with 80 mL of methylene chloride and collected, then passed through the Bond Elut extraction column, and vacuumized for 3 min at maximum negative pressure (−34 kPa). The Oasis MCX extraction column (Waters, MA, United States) was activated with 2 mL of methylene chloride before use. After the eluent completely passed through the Oasis MCX extraction column, the column was cleaned using 2 mL of methylene chloride, 2 mL of 0.1 mol/L hydrochloric acid–methanol (40:60, V/V), 2 mL of methanol, and 2 mL of water to elute impurities. Finally, the HAAs were eluted with 2 mL of 15% ammonium hydroxide–methanol (15:85, V/V). The eluent was blow-dried with nitrogen at 50°C and redissolved with 200 μL of methanol, and then a 0.20-μm microporous membrane was used for filtration analysis. The injection volume was 1 μL.

The chromatographic conditions for HAAs were as follows. The separation process was performed at 45°C at a flow rate of 0.4 mL/min using a ZORBAX Eclipse XDB-C_18_ (4.6 mm × 250 mm, 5.0 μm) reverse-phase analytical column (Agilent, United States) and solvent A (100% acetonitrile) and solvent B (0.01 mol/L phosphoric acid-triethylamine buffer solution, pH 3.6) solutions. The gradient program was: 0–0.1 min, 5% A; 0.1–3 min, 5–30% A; 3–9 min, 30–100% A, 9–12 min, 100% A; 12–13 min, 5% A.

The mass spectrometry conditions for HAAs were as follows. The electrospray was positive. The spectrometer was set to operate in MRM mode, with a capillary voltage of 3.5 kV, an extractor voltage of 3 V, and a collision gas (argon, 99.9% of purity) flow of 0.13 mL/min. The ion source temperature was 100°C. Desolvation gas (nitrogen, 99.9% of purity) temperature was 350°C, with a flow rate of 800 L/h. The cone (nitrogen, 99.9% of purity) flow rate was set to 50 L/h at a source temperature of 130°C.

#### Determination of Polyaromatic Hydrocarbons

A QuEChERS method based on Duedahl-Olesen et al. (26); Huang et al. (27) was used for the determination of PAHs from stir-frying mutton sao zi and with minor modifications. Initially, 2 g of homogenized sample (accurate to 0.01 g) was mixed in a 50-mL stoppered glass centrifuge tube, 1 g diatomite (alkaline) and 10 mL of n-hexane were added, vortex-shaken for 40 s, and then kept in an ultrasonic water bath at 40°C for 30 min. Then, after shaking for 1 min, centrifugation was performed at 4,500 r/min for 5 min at −4°C. The supernatant was collected and the bottom of the centrifuge tube was repeatedly extracted with 10 mL n-hexane once, and then the twice supernatant was collected in a 40-ml glass bottle. Nitrogen was blown (30°C) to remove the solvent, blowing to nearly dry. The 4 mL acetonitrile was added to the 40 mL glass bottle, vortex-mixed for 30 s, and then poured into the QuEChERS (P/N: MS-9PA1229) purification tube, vortex mixed for 30 s again, and centrifuged at 4,500 r/min for 3 min. The supernatant was taken in a 10-mL centrifuge tube, and the bottom of the centrifuge tube was repeated with 2 mL acetonitrile for extraction once, and the supernatant was collected in a 10-mL centrifuge tube again. The solvent was concentrated with nitrogen, and then made to a constant volume of 1 mL with acetonitrile. Finally, the solvent was filtered through a 0.22-μm PTFE-Q membrane filter and injected for GC-MS-TQ8040 (Shimadzu, Japan) analysis.

The PAHs were separated using a Rxi-5sil MS column (30 m × 0.25 mm, 0.25 μm) from Restek Inc. (Beijing, China). The initial oven temperature was maintained at 40°C, increased to 160°C at 30°C/min, then ramped to 320°C at 6°C/min, and finally increased to 320°C and held for 60 min. A solvent delay of 3.0 min was used. The injector was maintained at 300°C and 1 μL of extract was injected in splitless mode (100:1). Ultra-high purity helium (99.99%) was employed as carrier gas at a constant flow of 1.0 mL/min. The EI energy was 70 eV, transfer line and EI source temperature was set at 230°C, inlet temperature was set at 250°C, and interface temperature was 280°C.

### Statistical Analysis

The data were performed by IBM SPSS 24.0 and analyzed using analysis of variance (ANOVA) to determine significant differences (*P* < 0.05) among the treatments. The correlation analysis was performed in Origin 2021 using the correlation plot package. The mean values were presented and standard deviations were given as error bars in the figures. All the data are expressed as mean values ± standard deviations.

## Results and Discussion

### Changes of Basic Components

The basic components of stir-fried mutton sao zi developed in this study are presented in Table 1. Each processing stage of mutton was significantly different with respect to the cooking loss (*P* < 0.05), which showed that the water holding capacity of mutton protein was different in different stir-frying stages, which generally decreased with the prolongation of stir-frying time. This is because in the process of high-temperature stir-frying, on the one hand, the temperature will break the integrity and internal stability of myofibrillar protein, on the other hand, it will destroy the intermolecular force and the interaction between amino acids and surface water molecules, which will weaken the intermolecular force of myofibrillar protein (28). In general, the internal moisture of mutton was not retained and it was quickly lost with the extension of stir-frying time.

**TABLE 1:** Basic components characteristics of stir-frying mutton sao zi.

	Raw	SFRW 6 min	SFRW 7 min	SFRW 8 min	MSF 120 s	MSF 160 s	MSF 200 s
pH	5.5 ± 0.06^c^	5.65 ± 0.21^bc^	5.6 ± 0.07^bc^	5.84 ± 0.06^ab^	5.9 ± 0.09^a^	5.84 ± 0.08^ab^	5.94 ± 0.14^a^
Cooking loss (%)	0	17.32 ± 1.27^f^	21.08 ± 1.83^e^	23.67 ± 1.69^d^	27.59 ± 2.12^c^	31.64 ± 2.14^b^	33.18 ± 2.08^a^
L*	35.16 ± 0.67^c^	43.04 ± 0.86^b^	46.91 ± 1.36^a^	40.57 ± 0.94^b^	42.78 ± 1.32^b^	35.96 ± 0.96^c^	32.32 ± 0.98^d^
a*	16.07 ± 0.17^a^	9.85 ± 0.71^c^	8.49 ± 0.15^e^	10.46 ± 0.19^b^	7.73 ± 0.21^f^	9.2 ± 0.25^d^	8.63 ± 0.15^e^
b*	4.35 ± 0.25^d^	11.02 ± 0.66^ab^	10.5 ± 0.17^b^	11.01 ± 0.41^ab^	11.23 ± 0.33^a^	10.79 ± 0.14^ab^	9.14 ± 0.12^c^
Shear force/g	811.69 ± 30.71^h^	1624.83 ± 72.28^e^	2990.19 ± 122.15^d^	3119.77 ± 81.08^d^	4226.4 ± 201.91^c^	5351.12 ± 225^b^	7903.46 ± 206.36^a^
Hardness/g	963.69 ± 35.31^g^	2069.94 ± 76.69^f^	2585.03 ± 32.58^e^	3250.67 ± 121.99^d^	3839.35 ± 100.14^c^	4575.57 ± 147.7^b^	5326.66 ± 261.18^a^
Springiness/mm	0.83 ± 0.01^a^	0.54 ± 0.03^f^	0.58 ± 0.01^de^	0.65 ± 0.04^b^	0.63 ± 0.03^bc^	0.61 ± 0.02^cd^	0.56 ± 0.02^ef^
Gumminess/g	305.95 ± 15.76^f^	1616.86 ± 57.75^d^	1340.44 ± 89.74^e^	1483.97 ± 71.02^de^	2261.85 ± 140.81^c^	3389.78 ± 111.63^b^	5050.57 ± 239.81^a^
Chewiness/g	254.96 ± 14.00^f^	879.44 ± 71.79^d^	781.70 ± 47.65^e^	960.48 ± 93.96^d^	1414.61 ± 24.17^c^	2055.34 ± 18.41^b^	2825.37 ± 54.15^a^

The values denote mean ± standard deviation, n = 6. Different letters within the same line indicate significant differences (P < 0.05). SFRW, stir-frying to remove water; MSF, mixed stir-frying. There are two color systems, HunterLab and CIELAB. Color parameter values of the HunterLab system are represented by L, a, b, and color parameter values of the CIELAB system are represented by L*, a*, b*.

Many factors affect water retention of meat products, one of which is the pH value (28). The pH value of the sample at the MSF stage was significantly higher than that in the raw sample (*P* < 0.05), as shown in Table 1. After 8 min of stir-frying to remove water (SFRW), the pH value of the stir-fried sample increased by 0.04% compared with SFRW for 6 min. On the one hand, the original stable three-dimensional network structure of the protein is destroyed due to the breakage of amino acid side chains and peptide bonds (28). On the other hand, the protein in the mutton was decomposed to ammonia and amines with the high-temperature stir-frying condition and prolonged stir-frying time of the mutton sao zi, resulting in a rise in the pH value.

The textural attributes, hardness, and chewiness, in particular, were of great concern for the development of stir-frying mutton sao zi due to the poor tenderness of the water-holding capacity of the proteins. However, shear force, hardness, gumminess, and chewiness gradually increased with higher stir-frying temperature and longer time in different stir-frying stages (*P* < 0.05), mainly because the decrease in moisture content of mutton lead to the agglomeration of myofibrillar proteins during high-temperature stir-frying (29). The water-holding capacity of mutton protein weakens with the prolongation of stir-frying time, which leads to a decrease in the high springiness of raw meat as stir-frying progresses (28).

As shown in Table 1, the color change process of stir-fried mutton sao zi was characterized by measuring the L*, a*, and b* values. Between MSF120 and 200 s, the L* value is 42.78–32.32, which showed a decreasing trend. For the sample of MSF 120–200 s, the L* value is 42.78–32.32, showing a decreasing trend, while in the SFRW stage, it exhibited an upswing trend in 6–7 min as well as restoring a decline in 7–8 min. This is because the fresh mutton is red, and the denaturation of myoglobin during the stir-frying process could bring a variation to the color. The mutton sao zi was gradually darkened during the stir-frying, as the value a* decreased from MR. In addition, the value b* decreased slightly in SFRW 7 min but picked up in SFRW 8 min, exhibiting a slight decline in the MSF stage. Further prolonged stir-frying led to a burnt-taste phenomenon, which further promoted the change of values of L*, a*, and b* (8).

### Analysis of Thiobarbituric Acid Reactive Substances

**FIGURE 1:**
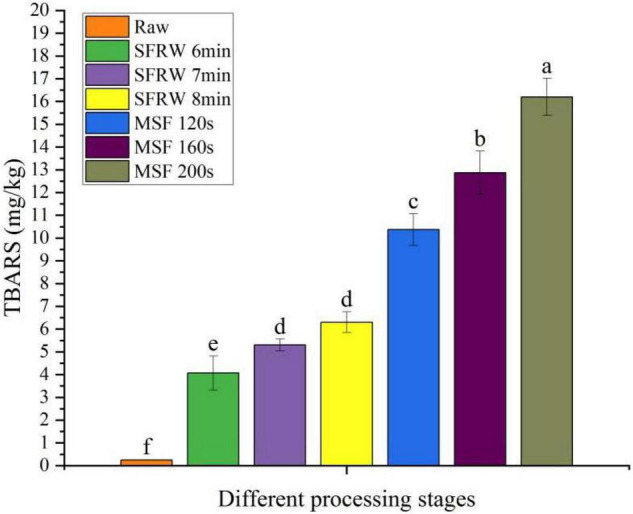
Thiobarbituric acid reactive substances (TBARS) content in different processing stages. Different small letters indicate a significant difference (*P* < 0.05).

The change of TBARS values during stir-frying of mutton sao zi process is shown in Figure 1. According to Cheng et al. (30), the lipid oxidation products are mainly produced under high-temperature conditions. Therefore, the TBARS value of the SFRW stage is lower than that of the MSF stage because the temperature of the SFRW stage is lower than 100°C. At the same time, the TBARS value in meat products was also related to the degree of oil absorption and oxidation during processing (31). The highest mean value of TBARS was determined to be 16.2 ± 0.82 mg MDA/kg in the MSF 200 s of stir-frying mutton sao zi. Since there was sheep oil in the MSF stage, high sheep oil uptake of stir-fried mutton made a valuable contribution toward lipid oxidation and thermal degradation. With the extension of stir-frying time, lipid oxidation was likely to increase. Compared with the SFRW stage, the high temperature and sheep oil of the MSF stage heightened the MDA content in the mutton sao zi, as shown in Figure 1. On prolonged stir-frying time, the TBARS value arose significantly (*P* < 0.05). Moreover, compared with the SFRW stage, a higher TBARS value was detected in the MSF stage as a result of added sheep oil as a heat transfer medium and a higher stir-frying temperature (>100°C).

### Analysis of Furosine

**FIGURE 2:**
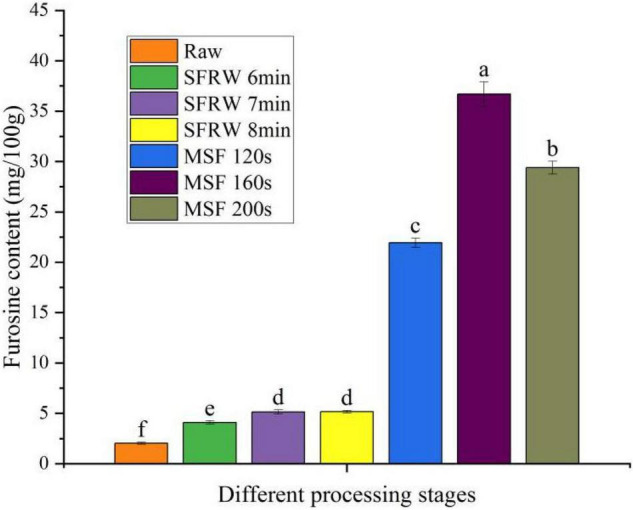
Furosine content in different processing stages. Different small letters indicate a significant difference (*P* < 0.05).

Furusine is often used to evaluate the severity of food heat treatment, and it is also the most specific and important indicator of early MR (32). As shown in Figure 2, furosine content in the MSF stage was significantly higher than that in SFRW stage, reached the maximum value at MSF 160 s (36.7 mg/100 g), and then dropped gradually (*P* < 0.05). At the same time, the furosine content was 2–3 times in the SFRW stage and 10–15 times in the MSF stage as that of raw meat. The furosine content dropped after MSF 160 s as a result of the degradation of furosine as the MR progressed. According to the Žilić et al. (33), the degradation of furosine will generate the intermediate and end products. Therefore, the Amadori products will significantly degrade when the furosine content is gradually reduced under high-temperature stir-frying conditions of mutton sao zi.

### Analysis of Fluorescent Compounds and Absorbance

**FIGURE 3:**
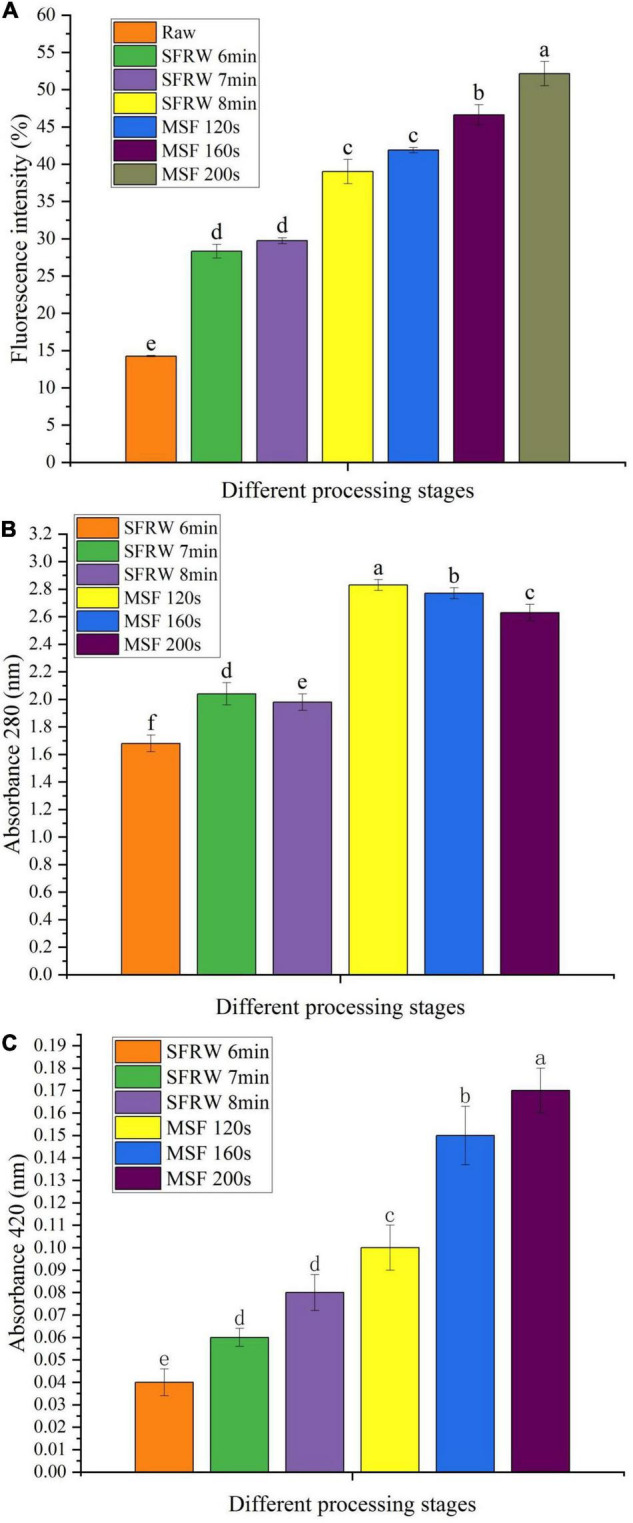
Fluorescence intensity **(A)** and absorbance at 280 nm **(B)** and 420 nm **(C)** of Tan sheep meat stir-fried at different processing stages. Different small letters indicate a significant difference (*P* < 0.05).

As the MR progresses in stir-frying mutton sao zi, colorless reductones and fluorescent substances were produced when Amadori products underwent fission and dehydration (34). Compared with cooking methods, such as stewing, steaming, or boiling, stir-frying process above 100°C will increase the product formation of MR in meat products (35). As shown in Figure 3A, the fluorescent compound gradually increased as stir-frying progressed. The fluorescence intensity was twice at 6–7 min and three times at 8 min in SFRW stage, and over three times in the MSF stage as that of raw meat (*P* < 0.05). In SFRW stage, the content of the fluorescent compound increased, but the fluorescence intensity became lower than that in the MSF stage. This could be because the reactants in SFRW stage flow out with water and diluted the fluorescent compound.

Figures 3B,C show the absorbance values of stir-frying mutton at 280 and 420 nm, respectively. The absorbance values at 280 nm were higher than 420 nm in different stir-frying processes. This result shows that there is a large amount of early MRP in stir-frying mutton sao zi.

The absorbance difference between SFRW and MSF stages at 280 nm was significant (*P* < 0.05), and the absorbance of 1.15 units increased significantly (*P* < 0.05) from the SFRW stage to the MSF stage during stir-frying (*P* < 0.05). Absorbance measurement at 280 nm decreases gradually after MSF 120 s and the difference was significant (*P* < 0.05), showing the decrease gradually of early MRP. Absorbance measurement at 420 nm increased gradually and the difference was significant (*P* < 0.05), indicating a higher browning rate during the whole stir-frying stage.

### Analysis of Nε-(1-Carboxymethyl)-L-Lysine and Nε-(1-Carboxyethyl)-L-Lysine

**FIGURE 4:**
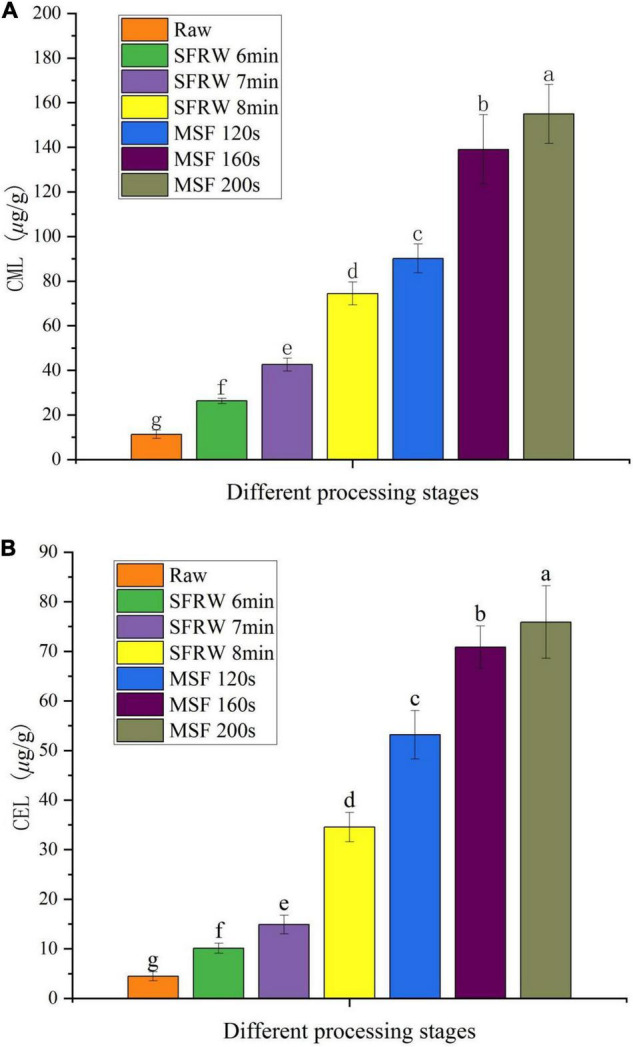
Nε-(1-carboxymethyl)-L-lysine (CML) **(A)** and Nε-(1-carboxyethyl)-L-lysine (CEL) **(B)** content in different processing stages. Different small letters indicate a significant difference (*P* < 0.05).

During the last stage of the MR, CML and CEL are two major AGEs produced, as well as existed in high temperature cooked processed meat products (36). As shown in Figures 4A,B, the CML and CEL content of mutton sao zi reached the maximum value in MSF 200 s, and a significant difference was observed in different stir-frying stages (*P* < 0.05). CML and CEL were detected in raw meat. This is due to the fact that, on the one hand, free CML and CEL are mainly produced by the body itself and during storage before the meat is thermally processed; on the other hand, CML and CEL production pathways include MR, thermal reactions, such as the auto-oxidative degradation of glucose, and the auto-oxidative degradation of lipids, and so a certain amount of CML and CEL can be detected in raw meat. Compared to raw meat, the content of CML and CEL increased by 6.6 times and 7.7 times at SFRW 8 min, and by 13.6 times and 17 times at MSF 200 s, respectively. In general, the content of CML and CEL continued to increase with the stir-frying time in mutton sao zi.

Wu et al. (31) also found that the lipid oxidation could form reactive carbonyl compounds, and then promote the generation of AGEs. Therefore, the high level of lipid oxidation is the main reason for the maximum CML and CEL content in the MSF stage. Based on the above results, a conclusion was drawn that CML and CEL were generated faster in the MSF stage than in the SFRW stage, and the high temperature processing accelerated the MR during stir-frying. Therefore, more active MR was observed in the MSF stage.

### Analysis of Acrylamides

**FIGURE 5:**
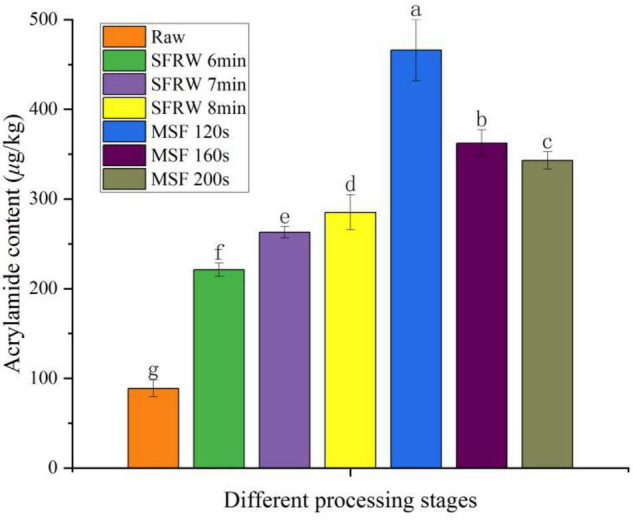
Acrylamide content in different processing stages. Different small letters indicate a significant difference (*P* < 0.05).

During high temperature cooking above 120°C, acrylamide (AA) is primarily produced from reducing sugars and free asparagine (31). As shown in Figure 5, it was obvious that the content of AA was significantly different at different stir-frying stages (*P* < 0.05). Starting from MSF 120 s, the AA content declined successively at MSF 160 s, MSF 200 s, SFRW 8 min, SFRW 7 min, and SFRW 6 min. That is to say, the content of AA in mutton sao zi did not increase with stir-frying time. Whereas, the AA content in the SFRW stage was lower than that in MSF stage. It was shown that the AA in stir-fried mutton sao zi mainly originated in the MSF stage in this study. That is because the stir-frying temperature in the SFRW stage is lower than 100°C, while the average stir-frying temperature in the MSF stage is higher than 120°C. According to Wang et al. (8), acrolein and acetaldehyde were the products through the amino group of AA crack in the process of stir-frying at a high-temperature. In this experiment, the AA content peaked at 465.89 μg/kg at MSF 120 s, which was a low level compared with Commission Regulation (37). The fact that AA content peaked at MSF 120 s and then declined can explain by the cracking reaction of AA. Since MSF is the most important stage for the stir-fried mutton sao zi to have the best quality, it is recommended the stir-frying time ends at some point after MSF 120 s. AA cannot be easily excreted from the body, but will be enriched in the human body with the continuous intake of AA. Therefore, it is essential to cut down the level of AA in high temperature processed meat products by optimizing processing parameters or reducing cooking temperature.

### Analysis of Heterocyclic Amines

Protein-rich foodstuffs can form carcinogenic HAAs during high-temperature cooking, such as frying and grilling (38). It was found that the influencing factors of different processing conditions on the production of HAAs in meat came in the order of time, temperature, and meat type (39). As shown in Table 2, MeIQx, 4,8-DiMeIQx, PhIP, Harman, and Norharman were detected from MSF 200 s to MSF 360 s, at the levels of 1.02–2.17, 0.59–1.34, 1.14–4.37, 0.27–0.54, and 2.05–3.19 μg/kg, respectively. The content of PhIP was the highest, which was the same case as cooking chicken at a high temperature reported by Aaslyng et al. (40). The amount of HAAs in processed meat can be significantly reduced by simply altering the cooking method without destroying the quality characteristics (1). All the 5 HAAs were detected after MSF 200 s, indicating that the best condition of stir-fried mutton sao zi appeared before MSF at 200 s. At the same time, this result is consistent with the previous experimental results for the flavor and sensory evaluation of stir-fried mutton sao zi, in which the optimum process parameter is MSF 160 s (6).

**TABLE 2:** Results of HAAs in mutton treated with stir-frying mutton sao.

HAAs	Raw	SFRW 6 min	SFRW 7 min	SFRW 8 min	MSF 120 s	MSF 160 s	MSF 200 s	MSF 240 s	MSF280 s	MSF 320 s	MSF 360 s
MeIQx	ND	ND	ND	ND	ND	ND	1.02 ± 0.07^c^	1.69 ± 0.14^b^	1.69 ± 0.14^b^	2.02 ± 0.05^a^	2.17 ± 0.09^a^
4,8-DiMeIQx	ND	ND	ND	ND	ND	ND	0.59 ± 0.04^d^	1.05 ± 0.07^c^	1.14 ± 0.09^bc^	1.24 ± 0.09^ab^	1.34 ± 0.09^a^
PhIP	ND	ND	ND	ND	ND	ND	1.14 ± 0.10^e^	2.60 ± 0.20^d^	2.29 ± 0.16^c^	3.50 ± 0.14^b^	4.37 ± 0.22^a^
Harman	ND	ND	ND	ND	ND	ND	0.27 ± 0.02^d^	0.54 ± 0.03^a^	0.47 ± 0.05^bc^	0.52 ± 0.04^ab^	0.46 ± 0.02^c^
Norharman	ND	ND	ND	ND	ND	ND	2.05 ± 0.12^b^	2.99 ± 0.19^a^	2.39 ± 0.24^b^	3.19 ± 0.23^a^	2.29 ± 0.15^b^

*ND = not detected, below limit of detection. The values denote mean ± standard deviation, n = 6. Different letters within the same line indicate significant differences (P < 0.05). SFRW, stir-frying to remove water; MSF, mixed stir-frying.*

### Analysis of Polyaromatic Hydrocarbons

The content of PAHs varies from different types, fat contents, and cooking methods (frying, roasting, grilling, boiling) of mutton products (1). The most important factor that affects the level of PAHs is the cooking time at a high temperature (41). As shown in Table 3, the 8 kinds of PAHs were detected in mutton sao zi of stir-frying process, which were naphthalene, phenanthrene, benz[a]anthracene, pyrene, chrysene, benzo[a]pyrene, benzo[k]fluoranthene, and dibenzo[a,h]anthracene. Only 2 PAHs, naphthalene and chrysene, were detected in the raw meat and in the SFRW stages. Naphthalene is not carcinogenic, but chrysene shows weak carcinogenicity. However, the content of chrysene in mutton cooked by steaming, boiling, and stewing is very low, which is obviously lower than that in mutton cooked by baking (42). Benzo[a]pyrene is a strong carcinogenic of thermal degradation of lipid and protein. When the stir-frying time exceeded the optimal processing time (MSF 160 s of 200 s, the content of benzo[a]pyrene peaked at 0.82 μg/kg, much lower than the maximum value of 5.0 μg/kg for heat-treated meat specified by the Commission of the European Communities (43). Therefore, stir-frying is as much recommendable as steaming, boiling, and stewing as a healthy cooking method.

**TABLE 3:** Results of PAHs in mutton treated with stir-frying.

No.	PAHs	Raw	SFRW 6 min	SFRW 7 min	SFRW 8 min	MSF 120 s	MSF 160 s	MSF 200 s	MSF 240 s	MSF 280 s	MSF 320 s	MSF 360 s
1	Naphthalene (–)	1.11 ± 0.07^f^	1.28 ± 0.14^e^	1.41 ± 0.06^e^	1.71 ± 0.05^e^	2.81 ± 0.17^d^	3.1 ± 0.27^e^	3.24 ± 0.19^d^	3.27 ± 0.26^d^	5.56 ± 0.19^c^	8.79 ± 2.22^b^	13.63 ± 0.52^a^
2	Phenanthrene (–)	ND	ND	ND	ND	ND	ND	ND	0.31 ± 0.04^c^	0.48 ± 0.07^b^	0.45 ± 0.02^b^	0.69 ± 0.08^a^
3	Pyrene (–)	ND	ND	ND	ND	0.38 ± 0.11^ef^	0.56 ± 0.06^ef^	0.94 ± 0.1^de^	1.32 ± 0.13^cd^	1.66 ± 0.09^c^	2.65 ± 0.07^b^	3.73 ± 0.95^a^
4	Benz[a]anthracene (+)	ND	ND	ND	ND	ND	0.19 ± 0.04^b^	0.18 ± 0.01^b^	0.18 ± 0.01^b^	0.21 ± 0.04^b^	0.31 ± 0.08^a^	0.18 ± 0.01^b^
5	Chrysene (+)	0.32 ± 0.03^h^	0.31 ± 0.12^h^	0.41 ± 0.03^g^	0.58 ± 0.15^f^	0.45 ± 0.09^g^	0.56 ± 0.08^f^	1.09 ± 0.03^e^	1.26 ± 0.15^de^	1.36 ± 0.06^c^	3.27 ± 0.11^b^	4.46 ± 0.19^a^
6	Benzo[k]fluoranthene (+ +)	ND	ND	ND	ND	0.3 ± 0.03^d^	0.36 ± 0.07^cd^	0.41 ± 0.05^cd^	0.44 ± 0.05^bc^	0.52 ± 0.05^b^	0.86 ± 0.19^a^	0.95 ± 0.03^a^
7	Benzo[a]pyrene (+ + +)	ND	ND	ND	ND	0.32 ± 0.01^e^	0.36 ± 0.01^d^	0.39 ± 0.07^c^	0.4 ± 0.03^c^	0.41 ± 0.03^c^	0.57 ± 0.05^b^	0.82 ± 0.18^a^
8	Dibenzo[a,h]anthracene (+ +)	ND	ND	ND	ND	0.38 ± 0.01^ef^	0.43 ± 0.06^e^	0.49 ± 0.05^e^	0.58 ± 0.12^d^	0.85 ± 0.11^c^	2.63 ± 0.14^b^	3.15 ± 0.13^a^

*“–” means not carcinogenic, “+” means weakly carcinogenic, “+ +” means carcinogenic, “+ + +” means strongly carcinogenic. ND = not detected, below limit of detection. The values denote mean ± standard deviation, n = 6. Different letters within the same line indicate significant differences (P < 0.05). SFRW, stir-frying to remove water; MSF, mixed stir-frying.*

### Correlation Between Maillard Reaction Products and Hazardous Substances

**FIGURE 6:**
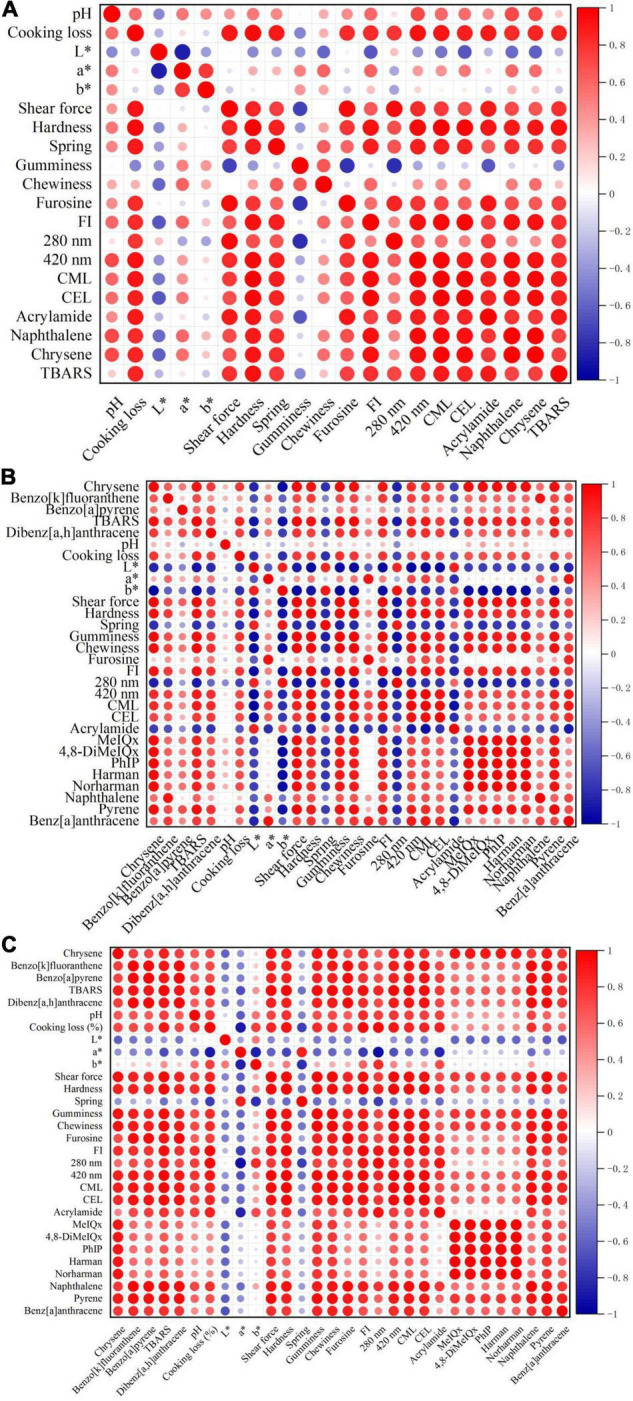
Correlation analysis among basic components, Maillard reaction products (MRP), and hazardous substances of stir-frying mutton sao zi. **(A)** Frying to remove water (SFRW) stage. **(B)** Mixed stir-frying (MSF) stage. **(C)** Whole stir-frying stage.

The basic components, MRP and hazardous substances of stir-frying mutton sao zi, were chosen to correlate with each other, as shown in Figure 6. The most important factor affecting MRP and potentially hazardous substances in processed meat products is temperature. As the temperature varies a lot in the two processing stages, the types and contents of MRP and hazardous substances are also different in SFRW and MSF stages, so they are separated for correlation analysis.

As shown in Figure 6A, it was observed that no trace of the 5 HAAs (4,8-DiMeIQx, MeIQx, Harman, PhIP, Norharman) and 6 PAHs (Phenanthrene, Pyrene, Benz[a]anthracene, Benzo[k]fluoranthene, Benzo[a]pyrene, Dibenzo[a,h]anthracene) were detected in the SFRW stage. The results indicated that pH, cooking loss, a*, b*, shear force, hardness, springiness, chewiness, furosine, FI, 280 nm, 420 nm, CML, CEL, AA, naphthalene, chrysene and TBARS of basic components, MRP, and hazardous substances had significant positive correlations with each other in the SFRW stage (*P* < 0.05). It was revealed that there was a strong correlation between MR and basic components, except for L* and gumminess during SFRW processing.

As shown in Figure 6B, a significant positive correlation was observed among pH, cooking loss, a*, shear force, hardness, gumminess, chewiness, furosine, FI, 420 nm, CML, CEL, TBARS, MeIQx, 4,8-DiMeIQx, PhIP, harman, norharman, naphthalene, pyrene, benz[a]anthracene, chrysene, benzo[k]fluoranthene, benzo[a]pyrene, and dibenzo[a,h]anthracene in MSF stage (*P* < 0.05). Besides, the L*, b*, springiness, 280 nm, and AA were significantly negatively correlated in the MSF stage. The phenomenon above indicates that the AA content does not gradually increase with the MR. Stir-frying can promote the formation of MRP in meat products, and it is a safe cooking method that barely produces harmful substances. The absorbance value at 280 nm does not show a positive correlation with MRP, which is consistent with the conclusions drawn from the research of Bombo Trevisan et al. (44) on different beef processing methods. It is indicated that changes in cooking temperature have no significant effect on the absorbance value at 280 nm.

The correlation among basic components, MRP, and hazardous substances in the stir-frying of mutton sao zi is displayed in Figure 6C. A significant positive correlation was observed among pH, cooking loss, b*, shear force, hardness, gumminess, chewiness, furosine, FI, 280 nm, 420 nm, CML, CEL, AA, MeIQx, 4,8-DiMeIQx, PhIP, harman, norharman, naphthalene, phenanthrene, pyrene, benz[a]anthracene, chrysene, benzo[k]fluoranthene, benzo[a]pyrene, dibenzo[a,h]anthracene, and TBARS during whole stir-frying stage (*P* < 0.05). The L*, b*, and springiness were significantly negatively correlated in the whole stir-frying stage. Therefore, no prediction of the correlation between MRP and hazardous substances could be made by using the indexes of L*, b*, and springiness in the whole stir-frying stage of mutton sao zi.

## Conclusion

This study focused on the progression of the MRPs and hazardous substances in different stir-frying procedures of mutton sao zi. As the early stage products pre-dominate the stir-fried mutton sao zi, the furosine content peaked at MSF 160 s and dropped thereafter. The CML and CEL, the advanced-stage products, increased further after MSF 160 s, suggesting that the MR was still going on. The AA content peaked at MSF 120 s and then dropped, HAAs were not detected before MSF 200 s, and PAHs ended up far lower than the maximum permissible value specified by the Commission of the European Communities. When the stir-frying time was extended by 200 s at the optimum process point (MSF 160 s), the content of hazardous substances increased but was still within the safety range for stir-frying mutton sao zi, validating that it is possible to prepare mutton products and preserve their nutritional qualities using the cooking methods of stir-frying.

## Data Availability Statement

The original contributions presented in this study are included in the article/supplementary material, further inquiries can be directed to the corresponding author.

## Author Contributions

SB: conceptualization, methodology and, writing – original draft, review and editing. LY: carried out the experiment, formal analysis, and investigation. YW: data curation. RL: review and editing, supervision, and funding acquisition. All authors contributed to the article and approved the submitted version.

## Conflict of Interest

The authors declare that the research was conducted in the absence of any commercial or financial relationships that could be construed as a potential conflict of interest.

## Publisher’s Note

All claims expressed in this article are solely those of the authors and do not necessarily represent those of their affiliated organizations, or those of the publisher, the editors and the reviewers. Any product that may be evaluated in this article, or claim that may be made by its manufacturer, is not guaranteed or endorsed by the publisher.
